# Supplementary material for: Titanocene Selenide Sulfides Revisited: Formation, Stabilities, and NMR Spectroscopic Properties
Source: Molecules. 2019 Jan 16;24(2):319. doi: 10.3390/molecules24020319 (PMC6358803; doi:10.3390/molecules24020319)
Supplement: Supplementary file 1 [file molecules-24-00319-s001.pdf]

Supporting Information

Titanocene Selenide Sulfides Revisited: Formation, Stabilities, and NMR Spectroscopic Properties

Heli Laasonen [1,2], Johanna Ikäheimonen [1,3], Mikko Suomela [1,4], J. Mikko Rautiainen [5] and Risto S. Laitinen [1,*]

[1] Laboratory of Inorganic Chemistry, Environmental and Chemical Engineering, University of Oulu, P.O. Box 3000, 90014 Oulu, Finland; heli.laasonen@valvira.fi (H.L.); johanna.ikaheimonen@outokumpu.com (J.I.); MikkoSuomela@eurofins.fi (M.S.)
[2] Department of Health, Legal Rights and Technologies, National Supervisory Authority for Welfare and Health (Valvira), P.O. Box 210, 00281 Helsinki, Finland
[3] Outokumpu Stainless Oy, Terästie 1, 95490 Tornio, Finland
[4] Eurofins Nab Labs Oy, Industry Services, Nuottasaarentie 17, 90400 Oulu, Finland
[5] Department of Chemistry, Nanoscience Centre, University of Jyväskylä, P.O. Box 35, 40014 Jyväskylä, Finland; j.mikko.rautiainen@jyu.fi
* Correspondence: risto.laitinen@oulu.fi; Tel.: +358-294-48-1611

Contents

1. Site occupation factors of selenium in chalcogen atom positions and interatomic distances

Table S1. The site occupancy factors of selenium in the disordered chalcogen atom sites of the crystals of phases *B*-*E*.
Table S2. The interatomic chalcogen-chalcogen and chalcogen-titanium distances.

2. The molecular composition of solid phases

Table S3. The composition of the solid solution of phases ***B***-***E*** based on the site occupancy factors of selenium in different chalcogen atom positions

3. Energetics of the twenty isomers of [$TiCp_2Se_xS_{5-x}$] (x = 0-5)

Table S4. Energy terms of atoms (in a.u.) used in the DLPNO-CCSD(T)/CBS enthalpy of formation calculations.
Table S5. Energy terms of atoms (in Hartree) used in the DLPNO-CCSD(T)/CBS enthalpy of formation calculations.
Table S6. The DLPNO-CCSD(T)/CBS formation enthalpies (298 K) of $TiCp_2Se_xS_{5-x}$ molecules calculated at non-relativistic $\Delta_f H_{nonrel.}$ and relativistic level $\Delta_f H$ with core-correlation and scalar relativistic [ΔE(C+R)] and spin-orbit [ΔE (SO)] energy corrections included.
Table S7. Isotropic ^{77}Se NMR shielding values calculated at PBE0/def2-TZVPP level of theory.

4. Quantitative details in syntheses

Table S8. The amounts of reagents and solvents in syntheses

5. PBE0/DEF2-TZVPP optimized geometries of [$TiCp_2Se_xS_{5-x}$] (x = 0-5)

1. Site occupation factors of selenium in chalcogen atom positions and interatomic distances

Table S1. The site occupancy factors of selenium in the disordered chalcogen atom sites of the crystals of phases ***B***-***E***.

Phase	E1	E2	E3	E4	E5
B	19.8(3)	51.5(3)	57.0(3)	43.0(3)	12.9(3)
C	23.1(4)	69.0(5)	84.6(6)	77.1(6)	34.5(5)
D [a]	45	82	82	71	32
E	82.1(5)	100.0	100.0	100.0	83.1(5)

[a] The data are taken from Pekonen, P.; Hiltunen, Y.; Laitinen, R. S.; Valkonen, J. ^{77}Se NMR Spectroscopic and X-ray Crystallographic Characterization of Bis(cyclopentadienyl)titanium Selenide Sulfides Mixtures [Ti(C$_5$H$_5$)$_2$Se$_x$S$_{5-x}$]. *Inorg. Chem.* **1991**, *30*, 1874-1878.

Table S2. The interatomic chalcogen-chalcogen and chalcogen-titanium distances.

Phase	Ti1-E1	Ti1-E5	E1-E2	E2-E3	E3-E4	E4-E5
B	2.4508(12)	2.4475(9)	2.1929(9)	2.2633(8)	2.2711(11)	2.1649(9)
C	2.4592(12)	2.4698(14)	2.2316(11)	2.3234(12)	2.3131(9)	2.2616(11)
D [a]	2.469(2)	2.469(2)	2.266(2)	2.312(1)	2.311(1)	2.239(2)
E	2.5191(13)	2.5545(13)	2.3207(15)	2.3373(11)	2.3353(10)	2.3199(15)

[a] The data are taken from Pekonen, P.; Hiltunen, Y.; Laitinen, R. S.; Valkonen, J. ^{77}Se NMR Spectroscopic and X-ray Crystallographic Characterization of Bis(cyclopentadienyl)titanium Selenide Sulfides Mixtures [Ti(C$_5$H$_5$)$_2$Se$_x$S$_{5-x}$]. *Inorg. Chem.* **1991**, *30*, 1874-1878.

2. The molecular composition of solid phases

All phases ***B***-***E*** are solid solutions of different [TiCp$_2$Se$_x$S$_{5-x}$] (x = 0-5) complexes. The identity of the complexes in different phases is based on the NMR assignment in the corresponding CS$_2$ solutions. The relative contents in the crystalline phases was estimated from the disorder scheme in chalcogen atom positions. However, in the solid state, the relative contents of individual [TiCp$_2$Se$_x$S$_{5-x}$] complexes can be different from those in solution because of the differing solubilities of the complexes. The results of the computations are shown in Table S3.

Table S3. The composition of the solid solution of phase ***B***-***E*** based on the site occupancy factors of selenium in different chalcogen atom positions (for numbering of chalcogen atom positions, see Figure 2 in the main text).

Complex		E1	E2	E3	E4	E5	Content [a]
(a) Phase ***B*** (Se:S = 1:4)							
[TiCp$_2$Se$_4$S] (**4$_1$**)	-Se-Se-Se-Se-S-	20	20	20	20	-	33
	-S-Se-Se-Se-Se-	-	13	13	13	13	
[TiCp$_2$SSe$_3$S] (**3$_6$**)	-S-Se-Se-Se-S-	-	1	1	1	-	1
[TiCp$_2$SSe$_2$S$_2$] (**2$_5$**)	-S-Se-Se-S-S-	-	15	15	-	-	23
	-S-S-Se-Se-S-	-	-	8	8		
[TiCp$_2$SSeS$_3$] (**1$_2$**)	-S-Se-S-S-S-	-	2	-	-	-	2
[TiCp$_2$S$_5$] (**0$_1$**) [b]	-S-S-S-S-S-	-	-	-	-	-	41
Total							100
Site occupancy factors of selenium	s.o.f(Calc.)	20	51	57	44	13	
(mol %)	s.o.f.(Exptl.) [c]	20	51	57	43	13	
(b) Phase ***C*** (Se:S = 2:3)							
[TeCp$_2$Se$_5$] (**5$_1$**)	-Se-Se-Se-Se-Se-	12	12	12	12	12	12
[TiCp$_2$Se$_4$S] (**4$_1$**)	-Se-Se-Se-Se-S-	8	8	8	8	-	25
	-S-Se-Se-Se-Se-	-	17	17	17	17	
[TiCp$_2$Se$_3$S$_2$] (**3$_1$**)	-Se-Se-Se-S-S-	3	3	3	-	-	9
	-S-S-Se-Se-Se-	-	-	6	6	6	

Table S1. *Cont.*

[$TiCp_2SSe_3S$] (**3**$_6$)	-S-Se-Se-Se-S-	-	18	18	18	-	18
[$TiCp_2SSe_2S_2$] (**2**$_5$)	-S-Se-Se-S-S-<br>-S-S-Se-Se-S-	-<br>-	6<br>-	6<br>15	-<br>15	-	21
[$TiCp_2SSeS_3$] (**1**$_2$)	-S-Se-S-S-S-<br>-S-S-S-Se-S	-<br>-	5<br>-	-<br>-	-<br>1	-<br>-	6
[$TiCp_2S_5$] (**0**$_1$) [b]	-S-S-S-S-S-	-	-	-	-	-	9
Total							100
Site occupancy factors of selenium (mol %)	s.o.f(Calc.)<br>s.o.f.(Exptl.) [c]	22<br>23	69<br>69	85<br>85	75<br>77	32<br>34	
Phase ***D*** (Se:S = 3:2)							
[$TeCp_2Se_5$] (**5**$_1$)	-Se-Se-Se-Se-Se-	25	25	25	25	25	25
[$TiCp_2Se_4S$] (**4**$_1$)	-Se-Se-Se-Se-S-<br>-S-Se-Se-Se-Se-	16<br>-	16<br>7	16<br>7	16<br>7	-<br>7	23
[$TiCp_2Se_3S_2$] (**3**$_1$)	-Se-Se-Se-S-S-	4	4	4	-	-	4
[$TiCp_2SSe_3S$] (**3**$_6$)	-S-Se-Se-Se-S-	-	24	24	24	-	24
[$TiCp_2SSe_2S_2$] (**2**$_5$)	-S-Se-Se-S-S-	-	6	6	-	-	6
[$TiCp_2S_5$] (**0**$_1$) [d]	-S-S-S-S-S-	-	-	-	-	-	18
Total							100
Site occupancy factors of selenium (mol %)	s.o.f(Calc.)<br>s.o.f.(Exptl.) [c,d]	45<br>45	82<br>82	82<br>82	72<br>71	35<br>32	
(d) Phase ***E*** (Se:S = 4:1)							
[$TeCp_2Se_5$] (**5**$_1$)	-Se-Se-Se-Se-Se-	65	65	65	65	65	65
[$TiCp_2Se_4S$] (**4**$_1$)	-Se-Se-Se-Se-S-<br>-S-Se-Se-Se-Se-	17<br>-	17<br>18	17<br>18	17<br>18	-<br>18	35
Total							100
Site occupancy factors of selenium (mol %)	s.o.f. (Calc.)<br>s.o.f. (Exptl.) [c]	82<br>82	100<br>100	100<br>100	100<br>100	83<br>83	

[a] in mol%. [b] Phases ***B-D*** also contain [$TiCp_2S_5$] the amount of which was estimated by completing the relative contents of the complexes to 100 %. [c] The experimental site occupancy factors of selenium in chalcogen atom positions have been taken from the refined crystal structures (see Table S1). The numerical values have been truncated to show only full integers. [d] Data are taken from Pekonen, P.; Hiltunen, Y.; Laitinen, R. S.; Valkonen, J. ^{77}Se NMR Spectroscopic and X-ray Crystallographic Characterization of Bis(cyclopentadienyl)titanium Selenide Sulfides Mixtures [$Ti(C_5H_5)_2Se_xS_{5-x}$]. *Inorg. Chem.* **1991**, *30*, 1874-1878.

3. Energetics of the twenty isomers of [$TiCp_2Se_xS_{5-x}$] (x = 0-5)

Table S4. Energy terms of atoms (in Hartree) used in the DLPNO-CCSD(T)/CBS enthalpy of formation calculations.

	**def2-TZVPP**		**def2-QZVPP**				
	**HF**	**CCSD(T)**	**HF**	**CCSD(T)**	**Total CBS**	**Δ*E*(C+R)**	**Δ*H*(0→298K)**
$\mathbf{0}_1$	-3220.81910	-3224.03662	-3220.90464	-3224.31691	-3224.47296	-12.02420	0.19777
$\mathbf{1}_1$	-5223.12788	-5226.43329	-5223.24930	-5226.80093	-5227.00005	-39.18928	0.19736
$\mathbf{1}_2$	-5223.13017	-5226.43503	-5223.25160	-5226.80172	-5227.00011	-39.18932	0.19735
$\mathbf{1}_3$	-5223.12991	-5226.43475	-5223.25134	-5226.80173	-5227.00034	-39.18951	0.19738
$\mathbf{2}_1$	-7225.44076	-7228.83271	-7225.59810	-7229.28723	-7229.52903	-66.35444	0.19703
$\mathbf{2}_2$	-7225.43897	-7228.83186	-7225.59630	-7229.28596	-7229.52746	-66.35454	0.19700
$\mathbf{2}_3$	-7225.43901	-7228.83185	-7225.59634	-7229.28586	-7229.52729	-66.35441	0.19702
$\mathbf{2}_4$	-7225.43630	-7228.83001	-7225.59360	-7229.28480	-7229.52684	-66.35434	0.19706
$\mathbf{2}_5$	-7225.44322	-7228.83445	-7225.60060	-7229.28818	-7229.52938	-66.35472	0.19705
$\mathbf{2}_6$	-7225.44141	-7228.83311	-7225.59876	-7229.28695	-7229.52825	-66.35435	0.19702
$\mathbf{3}_1$	-9227.75417	-9231.23239	-9227.94745	-9231.77393	-9232.05852	-93.51984	0.19671
$\mathbf{3}_2$	-9227.75231	-9231.23156	-9227.94554	-9231.77248	-9232.05665	-93.51953	0.19665
$\mathbf{3}_3$	-9227.74963	-9231.22975	-9227.94284	-9231.77119	-9232.05575	-93.51950	0.19668
$\mathbf{3}_4$	-9227.75237	-9231.23163	-9227.94564	-9231.77249	-9232.05657	-93.51978	0.19669
$\mathbf{3}_5$	-9227.74784	-9231.22871	-9227.94105	-9231.77007	-9232.05458	-93.51957	0.19669
$\mathbf{3}_6$	-9227.75666	-9231.23391	-9227.94999	-9231.77505	-9232.05932	-93.51990	0.19671
$\mathbf{4}_1$	11230.06785	11233.63236	11230.29707	11234.26080	11234.58808	-120.68506	0.19637
$\mathbf{4}_2$	11230.06327	11233.62977	11230.29243	11234.25802	11234.58520	-120.68490	0.19639
$\mathbf{4}_3$	11230.06299	11233.62914	11230.29212	11234.25779	11234.58529	-120.68469	0.19638
$\mathbf{5}_1$	13232.37891	13236.03047	13232.64400	13236.74626	13237.11664	-147.85022	0.19614
$\mathbf{S_2}$	-795.08548	-795.44394	-795.10877	-795.49526	-795.51923	-2.75729	0.00511
$\mathbf{Se_2}$	-4799.70605	-4800.23902	-4799.80138	-4800.46468	-4800.57435	-57.08803	0.004532

Table S5. Energy terms of atoms (in Hartree) used in the DLPNO-CCSD(T)/CBS enthalpy of formation calculations.

	def2-TZVPP		def2-QZVPP				
	**HF**	**CCSD(T)**	**HF**	**CCSD(T)**	**Total CBS**	**Δ*E*(SO)**	**Δ*E*(C+R)**
**Ti**	-848.39721	-848.72007	-848.413344	-848.753473	-848.768486	-0.00101	-4.51783
**C**	-37.69248	-37.78070	-37.693627	-37.786404	-37.789935	-0.00014	-0.05955
**H**	-0.49981	-	-0.499983	-	-0.500007	0.00000	-0.00001
**S**	-397.50200	-397.64607	-397.512996	-397.668363	-397.678243	-0.00089	-1.37824
**Se**	-2399.82719	-2400.05819	-2399.874606	-2400.167742	-2400.220298	-0.00431	-28.54398

Table S6. The DLPNO-CCSD(T)/CBS formation enthalpies (298 K) of $TiCp_2Se_xS_{5-x}$ molecules calculated at non-relativistic $\Delta_f H_{non-rel.}$ and relativistic level $\Delta_f H$ with core-correlation and scalar relativistic [Δ*E*(C+R)] and spin-orbit [Δ*E* (SO)] energy corrections included.

Complex	$\Delta_f H_{nonrel.}$	$\Delta_f H$	Complex	$\Delta_f H_{nonrel.}$	$\Delta_f H$
**0_1**	–25.2	–58.6	**3_1**	–38.9	–41.3
**1_1**	–26.2	–48.9	**3_2**	–34.2	–35.7
**1_2**	–26.4	–49.2	**3_3**	–31.7	–33.2
**1_3**	–26.9	–50.2	**3_4**	–33.9	–36.0
**2_1**	–31.9	–44.1	**3_5**	–28.6	–30.2
**2_2**	–27.9	–40.3	**3_6**	–41.0	–43.5
**2_3**	–27.4	–39.5	**4_1**	–46.2	–38.2
**2_4**	–26.1	–38.0	**4_2**	–38.6	–30.2
**2_5**	–32.8	–45.7	**4_3**	–38.9	–29.9
**2_6**	–29.9	–41.8	**5_1**	–50.6	–32.0

Table S7. Isotropic ^{77}Se-NMR shielding values calculated at PBE0/def2-TZVPP level of theory.

	Atom 1	Atom 2	Atom 3	Atom 4	Atom 5
**1_1**	657.8				
**1_2**		980.6			
**1_3**			1073.7		
**2_1**	701.4	1079.9			
**2_2**	628.7		1100.5		
**2_3**	645.9			979.3	
**2_4**	626.6				626.6
**2_5**		1043.7	1101.6		
**2_6**		1024.8		1024.8	
**3_1**	683.8	1151.9	1124.9		
**3_2**	696.4	1111.0		1019.2	
**3_3**	680.3	1064.1			614.8
**3_4**	619.0		1127.8	1041.8	
**3_5**	604.1		1135.3		604.1
**3_6**		1066.9	1143.0	1066.9	

Table S7. *Cont.*

**4**$_1$	676.4	1164.2	1159.6	1060.9	
**4**$_2$	664.3	1139.0	1158.7		590.3
**4**$_3$	665.0	1106.5		1106.5	665.0
**5**$_1$	648.0	1157.6	1190.3	1157.6	648.0

4. Quantitative details in syntheses

Table S8. The amounts of reagents and solvents in syntheses

Phase	Se:S	m_{Se} (g)	n_{Se} (mmol)	m_S (g)	n_S (mmol)
A				0.64	20.0
B	1 : 4	0.32	4.0	0.51	15.9
C	2 : 3	0.63	8.0	0.38	11.9
D	3 : 2	0.95	12.0	0.26	8.1
E	4 : 1	1.26	16.0	0.13	4.0
F		1.59	20.1		

The 0.1 M solution of lithium triethylhydridoborate in THF (8 ml, 8 mmol) was added into solid sulfur, selenium or their mixtures (see Table S8 for the quantities involved). The solution was stirred upon mild heating for 20 minutes, after which a solution of titanocene dichloride (1.00 g, 4.0 mmol) in 100 ml of THF was added dropwise during 30 minutes. The solution was filtered, the solvent THF was evaporated under dynamic vacuum, and the residue was extracted in 100 ml of CS_2. The NMR spectra were recorded from the saturated solutions thus obtained. The solid phases, which were used for crystal structure determinations, were obtained by recrystallization from these CS_2 solutions.

5. PBE0/def2-TZVPP optimized geometries of [$TiCp_2Se_xS_{5-x}$] (x = 0-5)

Cartesian *X*,*Y*,*Z* coordinates in Å units.

0$_1$ **[$Ti(C_5H_5)_2S_5$] (C_s)**

Ti	- 0.056104000	-0.931529000	0.000000000
S	0.727116000	0.470172000	1.797972000
S	-0.221400000	2.279067000	1.647879000
S	0.628288000	3.145016000	0.000000000
S	-0.221400000	2.279067000	-1.647879000
S	0.727116000	0.470172000	-1.797972000
C	-2.150980000	0.210661000	0.000000000
C	-2.121042000	-0.614048000	1.141448000
C	-2.062141000	-1.949046000	0.708911000
C	-2.062141000	-1.949046000	-0.708911000
C	-2.121042000	-0.614048000	-1.141448000
C	2.246694000	-1.545311000	0.000000000
C	1.655297000	-2.126736000	1.139452000
C	0.693649000	-3.059155000	0.705496000
C	0.693649000	-3.059155000	-0.705496000
C	1.655297000	-2.126736000	-1.139452000
H	-2.180086000	1.285377000	0.000000000
H	-2.117107000	-0.276305000	2.164112000
H	-2.045393000	-2.818902000	1.344464000

1$_2$ **[$Ti(C_5H_5)_2SSeS_3$] (C_1)**

Ti	1.177707000	0.030566000	-0.053434000
S	0.070822000	-2.075581000	0.378729000
S	-1.760345000	-2.069396000	-0.536954000
S	-2.894828000	-0.816258000	0.603772000
Se	-2.355255000	1.243869000	0.049356000
S	-0.423322000	1.443488000	1.053507000
C	2.271158000	-0.085424000	-2.142612000
C	1.983453000	1.278331000	-1.882160000
C	0.587158000	1.432768000	-1.884007000
C	0.010913000	0.173018000	-2.128287000
C	1.051736000	-0.764226000	-2.292922000
C	3.263082000	0.805529000	0.751191000
C	2.320331000	1.052055000	1.767280000
C	1.840221000	-0.191840000	2.228556000
C	2.493344000	-1.204805000	1.500535000
C	3.366851000	-0.591399000	0.582130000
H	3.253167000	-0.521536000	-2.223878000
H	2.704469000	2.065626000	-1.735795000

H	0.050136000	2.348490000	-1.702516000
H	-1.042192000	-0.041102000	-2.170391000
H	0.928425000	-1.818557000	-2.476623000
H	3.819243000	1.553527000	0.211488000
H	2.012894000	2.021905000	2.125432000
H	1.072760000	-0.340257000	2.970296000
H	2.348624000	-2.266519000	1.624463000
H	4.014857000	-1.105651000	-0.108134000

1_3 $[Ti(C_5H_5)_2S_2SeS_2]$ (C_s)

Ti	0.036826000	-1.252643000	0.000000000
S	-0.663005000	0.186140000	1.802393000
S	0.424775000	1.917759000	1.730605000
Se	-0.399612000	2.984633000	0.000000000
S	0.424775000	1.917759000	-1.730605000
S	-0.663005000	0.186140000	-1.802393000
C	1.981662000	-2.382977000	0.708950000
C	1.981662000	-2.382977000	-0.708950000
C	2.113836000	-1.053274000	-1.141361000
C	2.187831000	-0.231143000	0.000000000
C	2.113836000	-1.053274000	1.141361000
C	-0.838791000	-3.331586000	-0.705511000
C	-1.743234000	-2.343658000	-1.139740000
C	-2.298891000	-1.727531000	0.000000000
C	-1.743234000	-2.343658000	1.139740000
C	-0.838791000	-3.331586000	0.705511000
H	1.915074000	-3.250550000	1.344426000
H	1.915074000	-3.250550000	-1.344426000
H	2.129996000	-0.715538000	-2.164087000
H	2.271645000	0.840857000	0.000000000
H	2.129996000	-0.715538000	2.164087000
H	-0.266628000	-3.984694000	-1.342917000
H	-1.968346000	-2.097561000	-2.165621000
H	-2.987176000	-0.898301000	0.000000000
H	-1.968346000	-2.097561000	2.165621000
H	-0.266628000	-3.984694000	1.342917000

1_1 $[Ti(C_5H_5)_2SeS_4]$ (C_1)

Ti	1.027647000	-0.155964000	-0.055525000
S	-0.293118000	-1.543780000	1.394355000
S	-2.082478000	-1.944436000	0.475896000
S	-3.129930000	-0.193878000	0.621237000
S	-2.464895000	1.051682000	-0.854441000
Se	-0.568910000	1.828619000	-0.042854000
C	2.050327000	-1.613493000	-1.588879000
C	2.066642000	-0.317323000	-2.164405000
C	0.737772000	0.065683000	-2.406495000
C	-0.100677000	-0.986605000	-1.982153000
C	0.710614000	-2.025117000	-1.490349000
C	3.216787000	-0.314111000	0.807393000
C	3.042486000	1.004011000	0.336163000
C	2.063812000	1.624686000	1.137519000
C	1.624626000	0.689507000	2.095875000
C	2.340580000	-0.508397000	1.892953000
H	2.911363000	-2.195919000	-1.305214000
H	2.945212000	0.264986000	-2.388833000

2_1 $[Ti(C_5H_5)_2Se_2S_3]$ (C_1)

Ti	1.271188000	-0.099345000	-0.102873000
S	0.214454000	-1.903359000	1.096215000
S	-1.589802000	-2.352060000	0.232647000

H	0.410850000	1.003669000	-2.822978000
H	-1.175615000	-0.987900000	-2.017470000
H	0.362171000	-2.959376000	-1.083392000
H	3.911194000	-1.040025000	0.419091000
H	3.576276000	1.468756000	-0.476240000
H	1.727323000	2.646389000	1.048258000
H	0.844995000	0.847899000	2.822772000
H	2.229869000	-1.416790000	2.464109000

S	-2.839444000	-0.853718000	0.822928000
Se	-2.473207000	0.882356000	-0.477571000
Se	-0.479142000	1.668022000	0.433317000
C	2.346007000	-0.994573000	-1.995946000
C	2.067103000	0.369167000	-2.267918000
C	0.671555000	0.522783000	-2.320208000
C	0.087468000	-0.733956000	-2.069442000
C	1.121454000	-1.671598000	-1.878330000
C	3.270777000	1.064238000	0.368654000
C	2.308071000	1.540051000	1.279920000
C	1.934419000	0.470030000	2.119933000
C	2.675057000	-0.663821000	1.732262000
C	3.497378000	-0.300152000	0.648251000
H	3.324271000	-1.439098000	-1.917712000
H	2.794805000	1.146697000	-2.432117000
H	0.139383000	1.441500000	-2.500098000
H	-0.967137000	-0.939666000	-2.022542000
H	0.991530000	-2.717257000	-1.654528000
H	3.762034000	1.649191000	-0.390750000
H	1.934507000	2.550991000	1.335190000
H	1.181231000	0.501929000	2.890115000
H	2.618529000	-1.641406000	2.184535000
H	4.195552000	-0.946956000	0.144046000

2_2 $[Ti(C_5H_5)_2SeSSeS_2]$ (C_1)

Ti	1.313770000	-0.166707000	-0.043508000
S	-0.110091000	-1.618029000	1.238023000
S	-1.836107000	-1.987401000	0.198157000
Se	-2.997900000	-0.138195000	0.398540000
S	-2.070893000	1.256817000	-1.011297000
Se	-0.223686000	1.860450000	0.023911000
C	2.398743000	-1.517333000	-1.632464000
C	2.439005000	-0.183536000	-2.113130000
C	1.120898000	0.219565000	-2.381886000
C	0.265434000	-0.855257000	-2.063464000
C	1.055899000	-1.929348000	-1.613989000
C	3.440347000	-0.456878000	0.934596000
C	3.340733000	0.891521000	0.532107000
C	2.337105000	1.502701000	1.309265000
C	1.808815000	0.532030000	2.184471000
C	2.494741000	-0.678268000	1.954652000
H	3.247689000	-2.121247000	-1.357553000
H	3.325865000	0.410244000	-2.262275000
H	0.811776000	1.184556000	-2.746949000
H	-0.807423000	-0.849048000	-2.139052000
H	0.689822000	-2.887987000	-1.286812000
H	4.131653000	-1.185983000	0.546836000
H	3.938961000	1.380325000	-0.218846000
H	2.042470000	2.539955000	1.260148000
H	0.993857000	0.678913000	2.874166000

H	2.319464000	-1.612212000	2.465086000
H	-2.045393000	-2.818902000	-1.344464000
H	-2.117107000	-0.276305000	-2.164112000
H	2.981395000	-0.756984000	0.000000000
H	1.894661000	-1.894344000	2.165340000
H	0.084834000	-3.677922000	1.343157000
H	0.084834000	-3.677922000	-1.343157000
H	1.894661000	-1.894344000	-2.165340000

2_3 [Ti(C_5H_5)$_2$SeS$_2$SeS] (*C_1*)

Ti	-1.207850000	0.224946000	-0.068473000
S	0.386900000	1.488615000	1.205140000
Se	2.326904000	1.465151000	0.190104000
S	2.961960000	-0.614188000	0.514666000
S	1.940369000	-1.786689000	-0.800337000
Se	-0.035150000	-2.030878000	0.144727000
C	-1.969550000	1.685108000	-1.743800000
C	-2.288363000	0.367910000	-2.161490000
C	-1.085159000	-0.320148000	-2.381929000
C	-0.022089000	0.565726000	-2.103966000
C	-0.569603000	1.804115000	-1.724770000
C	-3.320091000	0.867109000	0.765604000
C	-3.379765000	-0.512695000	0.479048000
C	-2.501980000	-1.176018000	1.359341000
C	-1.890335000	-0.208313000	2.180323000
C	-2.399161000	1.055404000	1.814385000
H	-2.671791000	2.468660000	-1.511041000
H	-3.280637000	-0.031454000	-2.293338000
H	-0.985674000	-1.347903000	-2.688529000
H	1.025886000	0.328769000	-2.160793000
H	-0.012103000	2.680126000	-1.439488000
H	-3.890166000	1.640760000	0.279246000
H	-3.999748000	-0.986324000	-0.264211000
H	-2.347036000	-2.242780000	1.413986000
H	-1.133598000	-0.397486000	2.923782000
H	-2.123159000	2.000571000	2.254578000

2_4 [Ti(C_5H_5)$_2$SeS$_3$Se] (*C_s*)

Ti	0.175330000	-1.092623000	0.000000000
Se	-0.648195000	0.404514000	1.878018000
S	0.439793000	2.310592000	1.653608000
S	-0.405461000	3.158873000	0.000000000
S	0.439793000	2.310592000	-1.653608000
Se	-0.648195000	0.404514000	-1.878018000
C	2.178095000	-2.093671000	0.709386000
C	2.178095000	-2.093671000	-0.709386000
C	2.233203000	-0.758222000	-1.141792000
C	2.253562000	0.066755000	0.000000000
C	2.233203000	-0.758222000	1.141792000
C	-0.586324000	-3.204116000	0.705436000
C	-0.586324000	-3.204116000	-0.705436000
C	-1.548498000	-2.270793000	-1.140050000
C	-2.140275000	-1.689642000	0.000000000
C	-1.548498000	-2.270793000	1.140050000
H	2.166818000	-2.963957000	1.344564000
H	2.166818000	-2.963957000	-1.344564000
H	2.237215000	-0.420535000	-2.164418000
H	2.271092000	1.142055000	0.000000000
H	2.237215000	-0.420535000	2.164418000
H	0.020244000	-3.825384000	1.342770000
H	0.020244000	-3.825384000	-1.342770000
H	-1.800376000	-2.052775000	-2.166658000
H	-2.882360000	-0.907953000	0.000000000
H	-1.800376000	-2.052775000	2.166658000

2₅ [Ti(C₅H₅)₂SSe₂S₂] (*C*₁)

Ti	1.449246000	0.002671000	-0.033344000
S	0.195283000	-2.052009000	0.176010000
S	-1.558607000	-1.906477000	-0.866123000
Se	-2.816575000	-0.618355000	0.383179000
Se	-1.983913000	1.528659000	0.019643000
S	-0.098334000	1.434457000	1.125442000
C	2.617667000	-0.031393000	-2.084795000
C	2.426511000	1.325936000	-1.722177000
C	1.047788000	1.592055000	-1.757298000
C	0.385267000	0.406010000	-2.123759000
C	1.356050000	-0.594965000	-2.332824000
C	3.539585000	0.566738000	0.918987000
C	2.568901000	0.789211000	1.914108000
C	1.990329000	-0.454268000	2.245651000
C	2.611516000	-1.443861000	1.459285000
C	3.563031000	-0.814893000	0.633636000
H	3.565284000	-0.537466000	-2.168853000
H	3.200074000	2.038095000	-1.486616000
H	0.576271000	2.530294000	-1.518108000
H	-0.678946000	0.280292000	-2.215884000
H	1.158233000	-1.617357000	-2.608602000
H	4.165933000	1.318922000	0.469719000
H	2.307563000	1.743723000	2.342886000
H	1.182706000	-0.613129000	2.941104000
H	2.394910000	-2.500191000	1.484293000
H	4.207801000	-1.311248000	-0.072475000

2₆ [Ti(C₅H₅)₂SSeSSeS] (*C*ₛ)

Ti	1.510212971	0.064838241	0.000000000
S	0.167675479	-0.782967145	1.816542000
Se	-1.815806240	0.134627010	1.767851000
S	-2.688984101	-0.824621806	0.000000000
Se	-1.815806240	0.134627010	-1.767851000
S	0.167675479	-0.782967145	-1.816542000
C	2.444249310	2.112799662	0.709005000
C	2.444249310	2.112799662	-0.709005000
C	1.108327884	2.113813455	-1.141299000
C	0.282977160	2.106841970	0.000000000
C	1.108327884	2.113813455	1.141299000
C	3.672762028	-0.589664541	-0.705589000
C	2.786183885	-1.593412025	-1.139466000
C	2.231147437	-2.210171843	0.000000000
C	2.786183885	-1.593412025	1.139466000
C	3.672762028	-0.589664541	0.705589000
H	3.313933920	2.132355345	1.344638000
H	3.313933920	2.132355345	-1.344638000
H	0.771471384	2.095748278	-2.164182000
H	-0.792486543	2.089708290	0.000000000
H	0.771471384	2.095748278	2.164182000
H	4.263485057	0.046495134	-1.343119000
H	2.566153639	-1.843647209	-2.165191000
H	1.477251067	-2.980170414	0.000000000
H	2.566153639	-1.843647209	2.165191000
H	4.263485057	0.046495134	1.343119000

3₁ [Ti(C₅H₅)₂Se₃S₂] (*C*₁)

Ti	1.511787000	-0.131707000	-0.089687000
S	0.269646000	-1.986028000	0.814668000
S	-1.478274000	-2.255872000	-0.216599000
Se	-2.809401000	-0.683424000	0.528945000
Se	-2.116264000	1.257083000	-0.559123000
Se	-0.131909000	1.690618000	0.583747000
C	2.619130000	-0.878146000	-2.028270000
C	2.470865000	0.528218000	-2.136219000
C	1.098357000	0.812050000	-2.232241000
C	0.397880000	-0.408676000	-2.170783000
C	1.337623000	-1.451560000	-2.055912000
C	3.681050000	-0.575057000	0.724058000
C	3.543633000	0.826494000	0.635548000
C	2.561223000	1.225536000	1.562575000
C	2.084647000	0.071602000	2.219299000
C	2.782782000	-1.039007000	1.704451000
H	3.550548000	-1.415628000	-1.962501000
H	3.269391000	1.251221000	-2.164400000
H	0.655632000	1.790140000	-2.316239000
H	-0.671372000	-0.523275000	-2.195648000
H	1.108084000	-2.500918000	-1.975517000
H	4.369107000	-1.184529000	0.162772000
H	4.105101000	1.482993000	-0.007976000
H	2.243174000	2.239635000	1.749733000
H	1.295138000	0.040204000	2.952268000
H	2.648330000	-2.066068000	2.005126000

3₂ [Ti(C₅H₅)₂Se₂SSeS] (*C*₁)

Ti	1.416244000	-0.141268000	-0.104433000
S	0.096866000	-1.809503000	1.019887000
Se	-1.878690000	-1.985479000	0.093976000
S	-2.862605000	-0.148967000	0.772157000
Se	-2.128479000	1.503951000	-0.466812000
Se	-0.037857000	1.867745000	0.492859000
C	2.317390000	-1.177410000	-2.014274000
C	2.350027000	0.222911000	-2.237406000
C	1.024650000	0.682192000	-2.301270000
C	0.172910000	-0.425689000	-2.112977000
C	0.971318000	-1.572828000	-1.948579000
C	3.621400000	-0.632109000	0.585365000
C	3.522448000	0.769169000	0.454251000
C	2.617701000	1.232573000	1.429412000
C	2.147400000	0.119797000	2.155269000
C	2.773057000	-1.032144000	1.635793000
H	3.170817000	-1.830573000	-1.938125000
H	3.235458000	0.825726000	-2.354938000
H	0.710561000	1.702065000	-2.446627000
H	-0.902360000	-0.396046000	-2.090874000
H	0.611855000	-2.572804000	-1.773457000
H	4.251833000	-1.281691000	0.001898000
H	4.059632000	1.385264000	-0.247613000
H	2.349821000	2.263108000	1.604818000
H	1.406032000	0.141762000	2.937132000
H	2.621860000	-2.043560000	1.978316000

3₃ [Ti(C₅H₅)₂Se₂S₂Se] (*C*₁)

Ti	1.284293000	0.119236000	-0.182960000
Se	0.187631000	-2.065024000	0.527568000
S	-1.773488000	-2.164088000	-0.477761000
S	-2.920038000	-0.817269000	0.528846000
Se	-2.468335000	1.187194000	-0.252323000
Se	-0.462866000	1.644889000	0.844621000
C	2.349886000	-0.158572000	-2.262558000
C	1.989031000	1.207517000	-2.138401000
C	0.585570000	1.284203000	-2.132686000
C	0.078638000	-0.024615000	-2.228213000
C	1.168914000	-0.915393000	-2.319052000
C	3.333462000	1.050393000	0.511027000
C	2.396156000	1.364978000	1.514504000
C	1.971654000	0.157865000	2.109321000
C	2.652832000	-0.900253000	1.476179000
C	3.489516000	-0.351534000	0.483802000
H	3.354024000	-0.545509000	-2.318886000
H	2.666475000	2.044148000	-2.089527000
H	-0.000397000	2.183457000	-2.047526000
H	-0.961328000	-0.300386000	-2.224099000
H	1.102362000	-1.987741000	-2.395563000
H	3.853437000	1.759523000	-0.110915000
H	2.068307000	2.356352000	1.786520000
H	1.228240000	0.057860000	2.883255000
H	2.568359000	-1.947210000	1.724476000
H	4.149907000	-0.909481000	-0.158969000

3₄ [Ti(C₅H₅)₂SeSSe₂S] (*C*₁)

Ti	-1.471559000	0.189011000	-0.047827000
S	0.092105000	1.536839000	1.178217000
Se	1.983107000	1.700906000	0.084563000
Se	2.874239000	-0.432863000	0.368107000
S	1.714213000	-1.707884000	-0.979052000
Se	-0.178683000	-2.002383000	0.104936000
C	-2.387285000	1.597547000	-1.691984000
C	-2.621074000	0.261675000	-2.106859000
C	-1.377389000	-0.335462000	-2.365751000
C	-0.374351000	0.623684000	-2.110618000
C	-0.999978000	1.818008000	-1.709803000
C	-3.578830000	0.715808000	0.874547000
C	-3.581206000	-0.662136000	0.573009000
C	-2.637683000	-1.290085000	1.410033000
C	-2.043122000	-0.301703000	2.219455000
C	-2.628724000	0.938578000	1.890002000
H	-3.138065000	2.327283000	-1.436981000
H	-3.584843000	-0.208581000	-2.214002000
H	-1.211987000	-1.351555000	-2.682442000
H	0.686065000	0.462787000	-2.195954000
H	-0.499561000	2.731104000	-1.434979000
H	-4.205903000	1.465191000	0.421595000
H	-4.206348000	-1.158026000	-0.151084000
H	-2.428500000	-2.348441000	1.445036000
H	-1.250235000	-0.460677000	2.931707000
H	-2.384079000	1.891477000	2.331882000

3₅ [Ti(C₅H₅)₂SeSSeSSe] (*C*ₛ)

Ti	1.478382883	0.192090026	0.000000000
Se	-0.041608517	-0.572353510	1.883278000
S	-1.883876767	0.628199535	1.739582000
Se	-2.913937032	-0.210551925	0.000000000
S	-1.883876767	0.628199535	-1.739582000
Se	-0.041608517	-0.572353510	-1.883278000
C	2.555210137	2.155424780	0.709365000
C	2.555210137	2.155424780	-0.709365000
C	1.222646176	2.258855274	-1.141718000
C	0.398683508	2.307835483	0.000000000
C	1.222646176	2.258855274	1.141718000
C	3.558119362	-0.652164576	-0.705446000
C	2.587728553	-1.576751615	-1.140155000
C	1.983217550	-2.144993585	0.000000000
C	2.587728553	-1.576751615	1.140155000
C	3.558119362	-0.652164576	0.705446000
H	3.424555483	2.112271119	1.344545000
H	3.424555483	2.112271119	-1.344545000
H	0.885032817	2.275514744	-2.164257000
H	-0.675562677	2.361656397	0.000000000
H	0.885032817	2.275514744	2.164257000
H	4.202783427	-0.070495629	-1.342785000
H	2.360554679	-1.820047634	-2.166840000
H	1.174334138	-2.857419236	0.000000000
H	2.360554679	-1.820047634	2.166840000
H	4.202783427	-0.070495629	1.342785000

3₆ [Ti(C₅H₅)₂SSe₃S] (*C*ₛ)

Ti	1.794690391	-0.023290425	0.000000000
S	0.406952514	0.738432991	1.819605000
Se	-1.496652390	-0.337978171	1.842284000
Se	-2.568449789	0.600376957	0.000000000
Se	-1.496652390	-0.337978171	-1.842284000
S	0.406952514	0.738432991	-1.819605000
C	2.854375011	-2.010150212	-0.708976000
C	1.520960875	-2.091234339	-1.141184000
C	0.696335577	-2.132129541	0.000000000
C	1.520960875	-2.091234339	1.141184000
C	2.854375011	-2.010150212	0.708976000
C	3.910627218	0.767268037	0.705596000
C	3.910627218	0.767268037	-0.705596000
C	2.962490743	1.713023788	-1.139534000
C	2.369048543	2.293132200	0.000000000
C	2.962490743	1.713023788	1.139534000
H	3.723666729	-1.977034171	-1.344659000
H	1.183695127	-2.093614415	-2.164083000
H	-0.378576763	-2.175741264	0.000000000
H	1.183695127	-2.093614415	2.164083000
H	3.723666729	-1.977034171	1.344659000
H	4.539973775	0.169354823	1.343220000
H	4.539973775	0.169354823	-1.343220000
H	2.727787801	1.949273110	-2.165265000
H	1.569032835	3.015145532	0.000000000
H	2.727787801	1.949273110	2.165265000

4_1 [Ti(C_5H_5)$_2$$Se_4$S] ($C_1$)

Ti	1.639174000	-0.151519000	-0.075791000
S	0.251592000	-1.829592000	0.944105000
Se	-1.660137000	-2.027027000	-0.104908000
Se	-2.790029000	-0.115980000	0.593693000
Se	-1.837673000	1.627882000	-0.619256000
Se	0.191955000	1.868014000	0.499418000
C	2.622032000	-1.143304000	-1.969775000
C	2.673256000	0.262053000	-2.154047000
C	1.354926000	0.732351000	-2.264514000
C	0.488510000	-0.373408000	-2.141716000
C	1.272019000	-1.530278000	-1.973743000
C	3.802955000	-0.685575000	0.704933000
C	3.724378000	0.719696000	0.606988000
C	2.779015000	1.167334000	1.550394000
C	2.263233000	0.040950000	2.222729000
C	2.901798000	-1.103825000	1.702791000
H	3.467268000	-1.804558000	-1.873996000
H	3.566742000	0.861344000	-2.215725000
H	1.053862000	1.757794000	-2.397449000
H	-0.586673000	-0.336212000	-2.162782000
H	0.898377000	-2.531639000	-1.841270000
H	4.453995000	-1.326753000	0.135012000
H	4.300100000	1.348150000	-0.052010000
H	2.514883000	2.196122000	1.741142000
H	1.486861000	0.050600000	2.970113000
H	2.724653000	-2.122162000	2.010261000

4_2 [Ti(C_5H_5)$_2$$Se_3$SSe] ($C_1$)

Ti	1.527140000	0.065610000	-0.159162000
Se	0.253967000	-2.049826000	0.449408000
S	-1.638166000	-2.042355000	-0.678867000
Se	-2.874790000	-0.579719000	0.376363000
Se	-2.121190000	1.502746000	-0.345116000
Se	-0.130817000	1.697157000	0.855626000
C	2.621711000	-0.269736000	-2.213954000
C	2.399774000	1.123337000	-2.064446000
C	1.012366000	1.344764000	-2.091821000
C	0.376187000	0.097382000	-2.234751000
C	1.370974000	-0.898244000	-2.323360000
C	3.600763000	0.840606000	0.645556000
C	2.647931000	1.165729000	1.630914000
C	2.131351000	-0.038744000	2.153462000
C	2.772906000	-1.107018000	1.495145000
C	3.676390000	-0.565601000	0.559088000
H	3.581589000	-0.757585000	-2.256019000
H	3.158541000	1.883491000	-1.977182000
H	0.519397000	2.297307000	-1.996498000
H	-0.686288000	-0.070130000	-2.261156000
H	1.196791000	-1.955505000	-2.432518000
H	4.184334000	1.544839000	0.076743000
H	2.371075000	2.161982000	1.939248000
H	1.355975000	-0.127670000	2.896865000
H	2.618244000	-2.157213000	1.690303000
H	4.327989000	-1.132285000	-0.084897000

4_3 [Ti(C_5H_5)$_2$$Se_2SSe_2$] ($C_s$)

Ti	-0.194925000	1.452920000	0.000000000
Se	0.731167000	0.036452000	1.899231000
Se	-0.278921000	-2.060983000	1.775631000
S	0.689809000	-2.902940000	0.000000000
Se	-0.278921000	-2.060983000	-1.775631000
Se	0.731167000	0.036452000	-1.899231000
C	-2.259121000	2.322965000	0.709341000
C	-2.259121000	2.322965000	-0.709341000
C	-2.225510000	0.987000000	-1.141758000
C	-2.191020000	0.162577000	0.000000000
C	-2.225510000	0.987000000	1.141758000
C	0.418592000	3.615906000	-0.705522000
C	1.443754000	2.752825000	-1.139927000
C	2.073893000	2.213031000	0.000000000
C	1.443754000	2.752825000	1.139927000
C	0.418592000	3.615906000	0.705522000
H	-2.305264000	3.191839000	1.344821000
H	-2.305264000	3.191839000	-1.344821000
H	-2.206535000	0.649923000	-2.164367000
H	-2.139492000	-0.912121000	0.000000000
H	-2.206535000	0.649923000	2.164367000
H	-0.228797000	4.194166000	-1.343123000
H	1.710232000	2.553907000	-2.166479000
H	2.869042000	1.485339000	0.000000000
H	1.710232000	2.553907000	2.166479000
H	-0.228797000	4.194166000	1.343123000

5_1 [Ti(C_5H_5)$_2$$Se_5$] ($C_1$)

Ti	-0.163486000	1.667386000	0.000000000
Se	0.703187000	0.221200000	1.903207000
Se	-0.432482000	-1.814837000	1.853517000
Se	0.538003000	-2.833458000	0.000000000
Se	-0.432482000	-1.814837000	-1.853517000
Se	0.703187000	0.221200000	-1.903207000
C	-2.191627000	2.620152000	0.709267000
C	-2.191627000	2.620152000	-0.709267000
C	-2.209029000	1.284032000	-1.141625000
C	-2.204276000	0.458691000	0.000000000
C	-2.209029000	1.284032000	1.141625000
C	0.539636000	3.802308000	-0.705432000
C	1.528312000	2.898039000	-1.139820000
C	2.135479000	2.332431000	0.000000000
C	1.528312000	2.898039000	1.139820000
C	0.539636000	3.802308000	0.705432000
H	-2.204384000	3.490532000	1.344188000
H	-2.204384000	3.490532000	-1.344188000
H	-2.203452000	0.946332000	-2.164119000
H	-2.190324000	-0.617267000	0.000000000
H	-2.203452000	0.946332000	2.164119000
H	-0.083660000	4.406060000	-1.343152000
H	1.786834000	2.689125000	-2.166214000
H	2.901623000	1.574419000	0.000000000
H	1.786834000	2.689125000	2.166214000
H	-0.083660000	4.406060000	1.343152000
